# Osteoarthritis of the knee— biochemical aspect of applied therapies: a review

**DOI:** 10.17305/bjbms.2021.6489

**Published:** 2022-02-12

**Authors:** Anna Horecka, Anna Hordyjewska, Tomasz Blicharski, Jacek Kurzepa

**Affiliations:** 1 Department of Medical Chemistry, Medical University of Lublin, Lublin, Poland; 2 Department of Rehabilitation and Orthopaedics, Medical University of Lublin, Lublin, Poland

**Keywords:** Knee osteoarthritis, prostaglandin, nonsteroidal anti-inflammatory drugs, symptomatic slow-acting drugs for OA, corticosteroids, bisphosphonates

## Abstract

The most prevalent form of arthritis is osteoarthritis (OA) of the knee, which is characterized by a degeneration of articular cartilage resulting in the development of osteophytes, or bone spurs. Main goals of OA treatment are to reduce pain, slow the disease progression, and improve joint function and the quality of life. The purpose of this study was to verify all the therapies recommended by the European Society for Clinical and Economic Aspects of Osteoporosis and Osteoarthritis (ESCEO) from the biochemical point of view. Nonsteroidal anti-inflammatory drugs (NSAIDs) inhibit the synthesis of eicosanoids, whereas paracetamol prevents the production of prostaglandin (PG) by interacting with peroxidase (POX) site of the prostaglandin H2 synthase complex. Tramadol is an opioid that has a dual mechanism of action: It binds to the μ-opioid receptor and it inhibits serotonin and adrenaline. Corticosteroids, which are also prescribed for OA pain, inhibit the activity of phospholipase A2 and block the synthesis of arachidonate-derived eicosanoids. Symptomatic slow-acting drugs for osteoarthritis (SYSADOA) are drugs that are well tolerated by patients and help to restore proteoglycan matrix of the cartilage. These drugs include compounds that naturally build articular cartilage. The articular cartilage, as well as the bone located around the cartilage, is destroyed as osteoarthritis progresses. Thus, bisphosphonates, commonly used in the treatment of osteoporosis, were evaluated as potential therapy. However, there is no official recommendation for their use in therapy. The aim of the study was to analyze the biochemical mechanisms of principal drugs used for the treatment of knee OA. Therefore, a narrative review summarizing the current knowledge regarding the applied therapies was prepared.

## INTRODUCTION

Osteoarthritis (OA), the most common form of arthritis worldwide, is a degenerative arthropathy affecting all synovial joints, mainly cartilage and subchondral bone. The disease is also characterized by synovitis, meniscal degeneration, inflammation, and fibrosis of the infrapatellar fat pad [1,2]. OA is a leading cause of morbidity and chronic disability [3]. The occurrence of OA increases with age and about 35% of the population aged over 65 has symptomatic OA of the knee or hip [4]. The disease affects more than 250 million people worldwide [5]. The risk factors are obesity, female gender, genetic predisposition, and trauma [1]. OA of the knee is the most common form of OA and it is characterized by the degeneration of articular cartilage and bone, associated with inflammation [6,7]. The main reason is that the intrinsic repair mechanisms are insufficient and the imbalance between synthesis and degradation processes exists. The pathological changes occur slowly with mild to moderate symptoms. The number of lubricant molecules in the synovial membrane including hyaluronic acid secreted by fibroblast-like cells, and lubricin (proteoglycan 4; PRG4) secreted by the surface chondrocytes of the articular cartilage is decreased [7,8].

Advanced OA can be characterized by increased pain, swelling, stiffness and decreased mobility of the affected joint. The social costs of OA are very high and include surgery and rehabilitation, but also imply the loss of productivity [4,9]. The progressive deterioration of joint cartilages can result in a knee replacement surgery [1].

**FIGURE 1:**
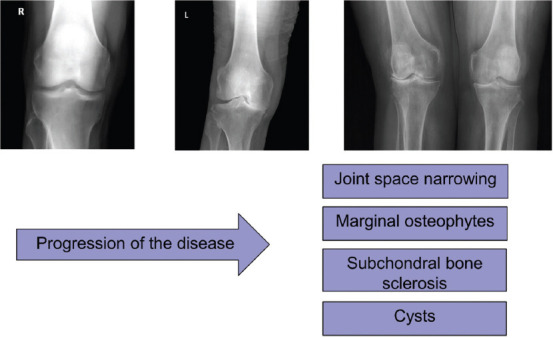
Progression of the knee OA. Radiological changes include mainly joint space narrowing. Marginal osteophytes, subchondral bone sclerosis and cysts indicate the presence of OA.

OA of the knee is diagnosed by the clinical and radiological assessment classified by the Kellgren and Lawrence score [10]. Radiological changes include mainly joint space narrowing. Marginal osteophytes, subchondral bone sclerosis, and cysts indicate the presence of OA (Figure 1). Radiographs assess the degree and location of OA. Symptoms associated with knee OA include: Morning stiffness <30 min, crepitus, instability, variety of motion deficit, varus or valgus deformity, joint-line tenderness, joint swelling/effusion, and absence of erythema [11]. The aim of the study is to analyze the biochemical mechanisms of principal drugs used for the treatment of knee OA. Therefore, a narrative review summarizing a current knowledge regarding the applied therapies was prepared.

We selected papers in English with relevance to knee OA and recommended therapies from the biochemical point of view. The bibliographical research was carried out on PubMed, Scopus and Web of Science databases, using keywords “knee osteoarthritis;” “inflammation in osteoarthritis;” “prostaglandin;” “nonsteroidal anti-inflammatory drugs;” “symptomatic slow-acting drugs for OA;” “corticosteroids;” “bisphosphonates;” “tramadol;” “paracetamol;” and “The European Society for Clinical and Economic Aspects of Osteoporosis and Osteoarthritis.” The word “Human” was used as a filter in PubMed. The most recent guidelines, protocols and recommendations for the treatment of knee OA were investigated. This research lead to 75 articles, and was completed at the end of August 2021.

## INFLAMMATION IN OA

Mechanical stress or trauma leads to the release of inflammatory mediators from all joint tissues involving mainly synovial membrane, but also cartilage, meniscus, subchondral bone, and infrapatellar fat pad [12,13]. Fibrinogen in synovial fluid activates inflammatory mediators such as tumor necrosis factor (TNF), interleukin-6 (IL-6), interleukin-1β (IL-1β), and vascular endothelial growth factor (VEGF) [14]. Intracellular proteins secreted as a result of stress or damage are able to send a signal to S-100 family of proteins (S100 A8 and S100 A9) in synovium and high mobility group box 1 protein (HMGB-1) [15,16]. Consequently, matrix metalloproteinases (MMPs): MMP-1, MMP-3, MMP-9, MMP-13, and IL-6 are released, contributing to the aggrecan and type-2 collagen catabolism. During the inflammatory process, basic calcium phosphate crystals and calcium pyrophosphate dehydrate crystals are observed in a synovial fluid [17]. Calcium pyrophosphate dehydrate stimulates chondrocytes, synovial cells to release nitric oxide (NO), IL-18, and IL-1β, prostaglandins and leukotrienes [17,18]. Inflammation also observed in THP-1 monocytes differentiated into macrophages designates a spontaneously immortalized monocyte-like cell line [19]. Prostaglandins (PG), mainly PGE2, are responsible for inflammation and pain. Nitric oxide contributes to the inflammatory reaction, pain, and apoptosis [20].

## OA TREATMENT

The goals for OA treatment are to reduce pain, improve joint function and quality of life, and slow the disease progression [21]. There is no appropriate therapy to prevent the OA progression [22]. OA treatment includes not only pharmacological, but also non-pharmacological approaches such as physical therapy, exercise, proper lifestyle, or patient education [23]. The European Society for Clinical and Economic Aspects of Osteoporosis and Osteoarthritis (ESCEO) recommends the use of symptomatic slow-acting drugs for OA (SYSADOA) as a first line pharmacological treatment, and paracetamol as a rescue medication when needed [5].

According to the World Health Organization (WHO) recommendation, paracetamol and nonsteroidal anti-inflammatory drugs (NSAIDs) are assigned to the first class of analgesics for mild pain, whereas tramadol should be administered at the second step for moderate pain [24].

**FIGURE 2:**
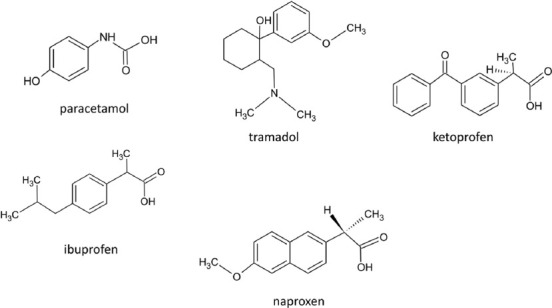
The chemical structures of the most known analgesic drugs.

The European League Against Rheumatism (EULAR) and the American College of Rheumatology (ACR) prepared recommendations for the pharmacological and non-pharmacological management of individuals with knee OA. They recommended paracetamol as first-line treatment, with topical agents such as topical NSAIDs [25-28]. Clinical trials demonstrated the safety and efficacy of different therapies in the knee OA treatment [17,18]. Drugs were divided into several categories. Fast-acting symptom modifying drugs included analgesics (e.g., paracetamol, tramadol), NSAIDs (e.g., ibuprofen, naproxen, and ketoprofen) (Figure 2), and corticosteroids administered mainly by intra-articular administration [4,29].

Unfortunately, long-term use of these drugs can induce a number of potentially serious side effects. The most common side effects of anti-inflammatory drugs are gastric discomfort, liver injury, cardiac, and renal adverse effects [30,31]. New treatment options are developed with the role blood-derived products, especially platelet-rich plasma (PRP) or bone marrow mesenchymal stromal cell treatment (BM-MSCs) [32,33].

### NSAIDs and prostaglandins

**FIGURE 3:**
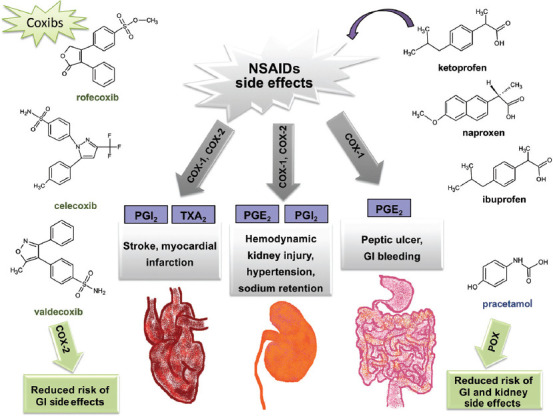
NSAIDs side effects and mechanism of their action. NSAIDs inhibit activity of both isoforms of COX. There are two types of NSAIDs: non-selective which inhibit both COX-1 and COX-2, such as ibuprofen, ketoprofen, diclofenac; and selective, mainly COX-2 inhibitors, such as coxibs. The reduced risk of the side effects is associated with the administration of paracetamol and coxibs [20]. The usage of coxibs (rofecoxib, celecoxib, and valdecoxib) is associated with a remarkably reduced risk of gastroduodenal lesions. An increased risk of acute myocardial infarction and kidney failure is associated with all NSAIDs, while the risk of a stroke is associated to the diclofenac and meloxicam usage, especially in the group of elderly.

Prostaglandins are eicosanoids, which are derivatives of the arachidonic acid (AC). They are key mediators of inflammation and the sensation of pain. Cyclooxygenases (COX-1 and COX-2) are the enzymes involved in their synthesis. PG stimulates pain receptors and contributes to the formation of fever and swelling. NSAIDs lead to a decreased synthesis of prostaglandins by inhibiting the activity of COX, which results in reduced pain perception, as well as a reduction in swelling and fever [34]. AC is a 20-carbon unit endogenous substance synthesized from linoleic acid or obtained from food. AC is found in membrane phospholipids and can be hydrolyzed by phospholipase A2 in a wide range of tissues [35]. Apart from the hydrolysis, it can be a substrate for COXs, lipoxygenases and cytochrome P450s. PG synthesis is regulated by the substrate availability and the level of COX expression. COX catalyses the transformation of AC to PGG2 and consequently to PGH2. There are two isoforms of COX. COX-1 is expressed continuously in most tissues, whereas COX-2 is enzyme induced by inflammation or carcinogenesis. COX-1 gene is located on the chromosome 9 and COX-2 gene is located on the chromosome 1 [36]. COX-1 produces prostaglandins which have a protective role for the gastric mucosa, and they are able to maintain the renal function and regulate the platelet aggregation [37]. NSAIDs inhibit activity of both COX isoforms. The inhibition of the production of prostaglandins by COX-1 blockade is the cause of the most of the adverse effects of NSAIDs. There are two types of NSAIDs: Non-selective which inhibit both COX-1 and COX-2, such as ibuprofen, ketoprofen, diclofenac; and selective, mainly COX-2 inhibitors, such as coxibs. The usage of coxibs (rofecoxib, celecoxib, and valdecoxib) is associated with a remarkably reduced risk of gastroduodenal lesions [38]. However, rofecoxib that contributed to acute coronary syndromes was withdrawn from the market in 2004 [30]. An increased risk of acute myocardial infarction and kidney failure is associated with all NSAIDs, while the risk of a stroke is linked to the diclofenac and meloxicam usage, especially in the group of elderly (Figure 3) [30].

Topical NSAIDs are suggested by the ESCEO for the patient who is still symptomatic after the background pharmacological therapy with SYSADOAs and a rescue analgesia with paracetamol. The short-term efficacy of topical NSAIDs in knee OA has been confirmed. Their administration is associated with the lower risk of adverse events in comparison to oral NSAIDs. For this reason, they are suggested for the patients aged ≥75 years. Diclofenac is the most effective among topical NSAIDs [5].

### Paracetamol

**FIGURE 4:**
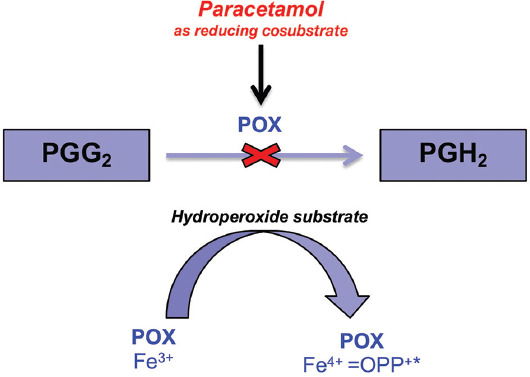
Mechanism of paracetamol action as reducing cosubstrate. The paracetamol mechanism of action is dependent on the COX having two sites: A COX site at the hydrophobic channel in the core of the enzyme and peroxidase (POX) site at the heme-containing active site at the protein surface. Paracetamol reduces the amount of the oxidized form by the action on the POX site. The first step of AC conversion to PGG2 is dependent on a tyrosine-385 radical (Tyr385*) at the COX site, generation of which is dependent on the availability of ferryl protoporphyrin IX radical cation (Fe 4+=OPP*+) located at the POX site. So, both sites cooperate to produce PGH2. Paracetamol inhibits this cooperation by acting as a reducing cosubstrate at the POX site.

Paracetamol is a p-aminophenol derivative that inhibits the activity of cyclooxygenase in the Central Nervous System (CNS). It has an antipyretic effect but it does not show anti-inflammatory and anti-aggregating properties. Opposite to NSAIDs, it does not irritate gastric mucosa and does not cause kidney damage [39]. The mechanism of action of paracetamol is dependent on the COX having two sites: A COX site at the hydrophobic channel in the core of the enzyme and peroxidase (POX) site at the heme-containing active site at the protein surface. Paracetamol reduces the amount of the oxidized form by the action on the POX site. The second theory is that paracetamol affects mainly CNS, where the COX-3 enzyme is present and inhibited by the drug. The first step of the AC conversion to PGG2 is dependent on a tyrosine-385 radical (Tyr385*) at the COX site, generation of which is dependent on the availability of ferryl protoporphyrin IX radical cation (Fe 4+=OPP*+) located at the POX site. Hence, both sites cooperate in the PGH2 production. Paracetamol inhibits this cooperation by acting as a reducing cosubstrate at the POX site (Figure 4) [39,40].

### Tramadol

Tramadol is a 2-(dimethyl amino)-methyl)-1-(3'-methoxyphenyl) cyclohexanol hydrochloride, that is the analogue of codeine. Commercially available tramadol is a racemic mixture of (+) tramadol and (−) tramadol enantiomers and is usually combined with paracetamol. It is an opioid acting on different receptors found in the brain, spinal cord and peripheral tissues [41]. Although tramadol has many side effects including nausea, dizziness, constipation, vomiting, somnolence, and headache, it is considered to be safe [42]. Tramadol acts in two different mechanisms. It binds to the μ-opioid receptor, its affinity is weak, and the drug is able to inhibit the reuptake of serotonin (+ enantiomer) and norepinephrine (+ enantiomer) [41,43]. Linking an agonist to an opioid receptor causes the inhibition of cyclic adenosine monophosphate (cAMP), decreases the flow of Ca2+ and increases the conductivity of potassium channels. These mechanisms represent a base for the analgesic action [44].

### Duloxetine

Duloxetine is a selective serotonin and norepinephrine reuptake inhibitor. The drug is mainly used as an antidepressant, but also in the treatment of chronic pain conditions such as peripheral neuropathic pain [45]. It was suggested that it can be used as a second-line agent for the treatment of knee OA in patients who did not respond or just partially responded to paracetamol and NSAIDs [46]. Adverse events related to duloxetine administration are: nausea, constipation, dry mouth, diarrhea, fatigue, dizziness, somnolence, and insomnia. Nevertheless, this drug is recommended because it significantly reduces pain, alleviates stiffness of the joints and improves their functioning [47].

### Sysadoa

**FIGURE 5:**
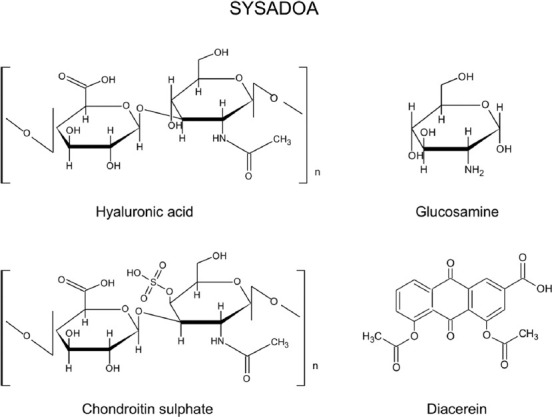
The chemical structures of SYSADOA.

Symptomatic slow-acting drugs for OA (SYSADOA) are much better tolerated by the patients than NSAIDs and they can improve clinical symptoms. Usually SYSADOA are prescribed together with analgesic and NSAIDs. They are known as chondroprotective substances that act slowly and bring relief to a patient [48]. SYSADOA can be administered systemically (e.g., chondroitin sulfate, glucosamine sulfate, and diacerhein), as well as intra-articularly (e.g., hyaluronic acid) (Figure 5) [48].

**FIGURE 6:**
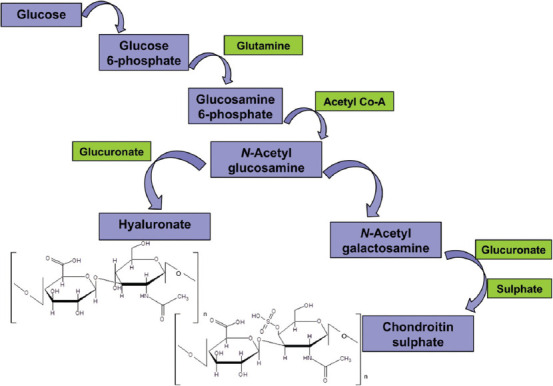
Biosynthetic pathway of glycosaminoglycans. Chondroitin sulfate, glucosamine sulfate, and hyaluronic acid are the precursors of cartilage matrix.

Chondroitin sulfate, glucosamine sulfate, and hyaluronic acid are the precursors of cartilage matrix, physiologically produced in the human body (Figure 6), whereas diacerhein is a cytokine modulator. Both glucosamine and chondroitin are the substrates for proteoglycan synthesis, essential for the cartilage integrity [49,50]. After the administration, they are absorbed and reach the joints improving their function [48].

Glucosamine (GS), the main long-chain glycosaminoglycan, is a water soluble amino monosaccharide synthetized by chondrocytes. GS is the most abundant monosaccharide, mainly located in articular cartilage [51]. Glucosamine sulfate is the most known and used formula in OA patients [52,53]. It shows anti-inflammatory activity and helps to restore proteoglycan matrix of the cartilage. GS decreases PGE2 production and inhibits nuclear factor kappa B (NFĸB) pathway leading to pro-inflammatory cascade in synovial fluid [54]. IL-1β, a pro-inflammatory cytokine, is released in synovial fluid and activates COX-2 and matrix metalloproteinases. GS is able to reduce COX-2 activity, nitric oxide synthase (iNOS), matrix metalloproteinases, thereby controlling the inflammatory cascade [55,56]. GS can be used long term, with adverse events comparable to placebo. ESCEO suggested that only patented crystalline glucosamine sulfate is able to reach a proper concentration in plasma corresponding to the clinical efficacy and bioavailability [49].

Chondroitin sulfate, the main component of extracellular matrix (ECM) is an unbranched complex polysaccharide consisting of glucuronic acid and N-acetyl-D-galactosamine. Its crucial role is to create osmotic pressure [51]. The European Medicine Agency (EMA) recommended chondroitin sulfate as a biologically active molecule [49]. It is anti-inflammatory agent, able to stimulate the synthesis of proteoglycan and hyaluronic acid, as well as inhibit the synthesis of proteolytic enzymes and NF-κB contributing to the cartilage damage [18]. In chondrocytes, pro-inflammatory cytokines as IL-1 and TNF, and pro-inflammatory enzymes, such as phospholipase A2 (PLA2), COX-2 and iNOS-2 are decreased [57].

ACR guidelines do not recommend the usage of chondroitin sulfate, glucosamine, and combination products including two of them for knee OA, but they are recommended for hand OA [26]. Hyaluronic acid (HA) is a linear polysaccharide of synovial fluid and cartilage secreted by fibroblast-like cells in the synovial membrane. The synovial fluid is a viscous fluid that has lubrication, metabolic, and regulatory functions [58]. HA supplements decrease OA pain by lubricating knee joints and provide an opportunity to treat symptomatic OA and improve its function. HA is composed of D-glucuronic acid and D-acetylglucosamine. Its powerful activity includes the inhibition of MMPs activity, chemokines and prostaglandins. Intra-articular injections restore viscoelastic properties of articular cartilage and they are well tolerated [22,27]. HA decreases the number of inflammatory molecules such as PGE2, IL-1, and IL-6. However, even though several injections of HA contribute to the decrease of short-term pain, it is postulated that in case of future joint replacement, HA usage may increase the joint infection [1]. New treatment options with highly crosslinked hyaluronic acid of different molecular weight and combinations of substances were proposed. Based on the molecular weight, HA exists in three categories (high ≥3000 kDa, moderate 1500–3000 kDa, and low ≤1500 kDa) [29]. High-molecular-weight intra-articular hyaluronic acids (HMW IA-HA) have better chondro-protective and anti-inflammatory properties [30]. Combining platelet-rich plasma (PRP) with HA resulted in better outcomes in comparison to HA alone [59]. There are different sources of HA: Biological fermentation-derived HA and avian-derived HA [28]. It was suggested that HMW IA-HA and those biological fermentation-derived HA provide better efficacy and safety [60]. Diacerein is an anthraquinone derivative with anti-inflammatory, analgesic and pro-anabolic properties for cartilage. The active metabolite of the drug is rhein. Positive effect of diacerein for subchondral bone was also observed [48]. It stimulates collagen, proteoglycan, and hyaluronic acid synthesis. Diacerein is able to reduce the inflammation by the IL-1β inhibition [61]. It differs from NSAIDs as it does not inhibit prostaglandin synthesis or affect their concentration. Diacerein is believed to work by reducing the action of interleukin-1β, a protein involved in the destruction of articular cartilage and synovial fluid. Although diacerein slows down the progression of the disease visible on the radiograph, no effect on signs and symptoms was observed [62]. Unfortunately, the most common adverse events are loose stools or diarrhea [63].

### Corticosteroids (CS)

Corticosteroids in OA are not orally administrated. Intra-articular corticosteroids (IACS) (e.g., triamcinolone) are short-term pain relief drugs which decrease the synthesis of pro-inflammatory mediators such as prostaglandins and leukotrienes, neutrophil superoxide, and MMPs. CS inhibit the activity of PLA2, and as a result, the synthesis of arachidonate-derived eicosanoids is blocked [64]. When it comes to the synovitis diagnosis, it is important to find a proper group of patients who respond to the IACS treatment [65]. Synovitis can be confirmed by arthrocentesis, ultrasonography, MRI, or by clinical diagnosis confirming redness, warmness, pain, and the presence of swollen joint with effusion [65]. However, the chronic use of the IA corticosteroids is not recommended because of the adverse events on the articular cartilage and because it contributes to the OA progression. IACS is prescribed for patients who do not respond to the standard therapy [65].

### Bisphosphonates (BPS)

Bisphosphonates are derivatives of inorganic pyrophosphate (PPi), able to bind to the hydroxyapatite crystals in a bone. It was reported that not only cartilage is involved in the development of OA, but the subchondral bone seems to play a role in the pathogenesis of the disease [66]. For this reason, BPs were evaluated as potential therapy for the knee OA [67]. BPs are the most common anti-osteoporotic drugs because of their ability to inhibit the bone resorption by osteoclast apoptosis [48]. It was reported that they have an analgesic effect on the OA. Subchondral bone lesions are visible in magnetic resonance imaging (MRI) in many patients with OA [68]. BPs delay and reverse pathological changes occurred in the bone and cartilage, and have a pain-relieving effect in animal model [69]. They can inhibit MMPs expression [70]. However, there is no official statement for the bisphosphonates usage in therapy. ESCEO does not recommend them for the treatment of knee OA [71].

There are clinical trials confirming that risedronate showed positive effects on the joint structure in OA, but not on the decrease of the disease progression [62]. The effects of BPs in the treatment of OA pain was investigated by a meta-analysis by Davis et al. evaluating 13 clinical trials [72]. There is an insufficient evidence that BPs are useful in the pain treatment [72]. The effectiveness of intramuscular clodronate in reducing pain and bone marrow edema in knee osteoarthritis was evaluated in a clinical trial. Results confirmed therapeutic effect directly proportional to the treatment duration and pain reduction coupled with a good safety profile [73]. Clodronate has an anti-inflammatory effect on OA cartilage and is well-tolerated. It inhibits the release of inflammatory cytokines ((IL)-1β, IL-6, TNF-γ) [74]. BPs are widely used for the treatment of hypercalcemia, multiple myeloma, post-menopausal osteoporosis as oral, intravenous (i.v.), and intramuscular (i.m.) formulations [67,72,75].

## CONCLUSION

**FIGURE 7:**
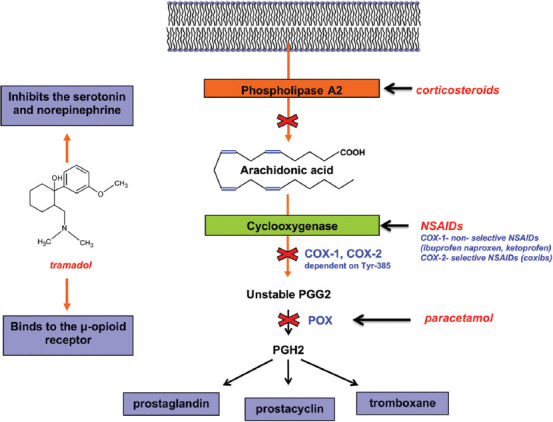
Mechanism of action of fast-acting symptom modifying drugs including analgesics (e.g., paracetamol and tramadol), nonsteroidal anti-inflammatory drugs (NSAIDs) (e.g., ibuprofen, naproxen, and ketoprofen), coxibs and corticosteroids. NSAIDs inhibit the synthesis of eicosanoids. Paracetamol prevents the production of prostaglandins by the POX interaction. Tramadol is an opioid that has a dual mechanism of action - one by binding to the μ-opioid receptor and the other by inhibiting serotonin and adrenaline. Corticosteroids inhibit the activity of phospholipase A2 and block the synthesis of arachidonate-derived eicosanoids.

There were numerous clinical studies conducted, supporting the beneficial effects of mentioned drugs for knee OA patients. The aim of this study was to evaluate the biochemical aspect of the recommended therapies and the action mechanisms of all individual groups of drugs. The best known drugs are NSAIDs, which inhibit the synthesis of eicosanoids, and paracetamol, which prevents the production of prostaglandins by interacting with POX. Tramadol is an opioid that has a dual mechanism of action - one by binding to the μ-opioid receptor and the other by inhibiting serotonin and adrenaline. Corticosteroids inhibit the activity of phospholipase A2 and block the synthesis of arachidonate-derived eicosanoids (Figure 7). SYSADOA are drugs that are very well tolerated by patients. These include compounds that naturally build articular cartilage. Not only is the articular cartilage destroyed during the progress of osteoarthritis, but also the bone located around the cartilage. Thus, bisphosphonates, commonly used in the treatment of osteoporosis, were evaluated as potential therapy. However, there is no official recommendation to use them in therapy. All the clinical recommendations were previously provided by ESCEO, ACR, or EULAR societies. Physician should take into consideration the preferences of the patient, as well as the patient’s medical status, and choose the most appropriate management plan. There is no single right approach of the treatment.

## Figures and Tables

**FIGURE S1:**
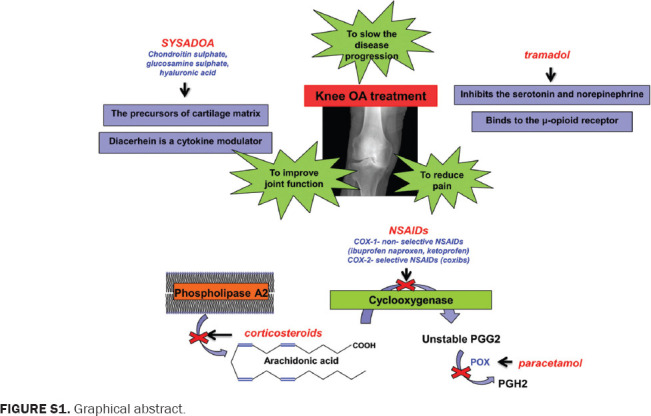
Graphical abstract.
